# Tissue-specific untargeted ^1^H-NMR metabolomics reveals no adverse metabolic effects of daily intrabuccal cannabidiol (CBD) in healthy mice

**DOI:** 10.3389/fphar.2026.1896842

**Published:** 2026-08-20

**Authors:** M. V. Masiphephethu, S. Bhengu, K. Venter, Y. Lemmer, S. Mason

**Affiliations:** 1 Biomedical and Molecular Metabolism (BioMMet) Research Group, Faculty of Natural and Agricultural Sciences, North-West University, Potchefstroom, South Africa; 2 Preclinical Drug Development Platform, Faculty of Health Sciences, North-West University, Potchefstroom, South Africa; 3 Future Production and Chemicals, Council for Scientific and Industrial Research (CSIR), Pretoria, South Africa

**Keywords:** brain, cannabidiol (CBD), kidneys, liver, metabolism, mice, proton magnetic resonance (^1^H-NMR), untargeted metabolomics

## Abstract

**Background:**

Cannabidiol (CBD) has attracted renewed scientific attention for its therapeutic potential in human metabolism. However, the metabolic effects of CBD treatment remain incompletely understood.

**Methods:**

This study investigated tissue-specific metabolic alterations in the brain, liver, and kidneys of Kramnik mice after 2 weeks of daily CBD treatment using an untargeted proton magnetic resonance (^1^H-NMR) metabolomics approach.

**Results:**

Unsupervised principal component analysis (PCA) revealed minimal biological variation between the CBD-treated and control groups. The most notable, albeit minor, changes were observed in the brain and were associated with decreased signaling and neuroenergetics. The liver showed changes in sugar metabolism, and the kidneys showed only minor changes in phenylalanine levels.

**Discussion:**

Collectively, our results suggest a more metabolically balanced state in the mice. These data support the adjunctive use of CBD, as no adverse metabolic effects were observed in the brain, liver, or kidneys.

## 1 Introduction

Cannabis (*Cannabis sativa*) is a medicinal plant used for thousands of years, with renewed focus on one of its constituents–cannabidiol (CBD) (Russo, 2007). The therapeutic effects of cannabis have been documented throughout ancient Asian medicinal history for anodyne, soothing, and sedative effects (Russo, 2007; Li, 1974). CBD is known to have non-psychoactive effects on the endocannabinoid system in humans, unlike the psychoactive component tetrahydrocannabinol (THC) (de Oliveira Carvalho et al., 2024). CBD is known to interact with various molecular targets involved in redox control, calcium signaling, and mitochondrial function (Bih et al., 2015; Atalay et al., 2019). Organs with high metabolic demand, such as the liver, kidney, and brain, are highly susceptible to oxidative stress due to increased oxygen use and dense mitochondrial populations (Betteridge, 2000; Murphy, 2009). Furthermore, even minor disturbances in redox balance and energy metabolism in these tissues can have serious physiological implications for the host. Therefore, it is important to determine whether CBD can alter oxidative stress pathways and cellular bioenergetics, thereby influencing metabolic homeostasis in *in vivo* studies.

Metabolomics provides a scientific approach for assessing and identifying biological changes induced by pharmacological therapies by profiling low-molecular-weight metabolites and detecting downstream functional alterations that reflect pathway activity, mitochondrial efficiency, and redox state (Nicholson et al., 1999; Patti et al., 2012). In this study, proton nuclear magnetic resonance (^1^H-NMR) spectroscopy was used as the analytical method because of its high reproducibility, quantitative robustness, and minimal sample preparation requirements for detecting primary metabolites, such as amino acids and organic acids (Emwas, 2015; Wishart, 2019). Therefore, this study aims to investigate tissue-specific metabolic alterations in the liver, kidney, and brain after 2 weeks of CBD administration in healthy Kramnik mice.

## 2 Methods

### 2.1 Study design and animal husbandry

A total of 10 Kramnik (C3HeB/FeJ) mice (JAX stock # 000658; The Jackson Laboratory), both male and female, were bred and raised to adulthood (8 weeks old), as described by Omotayo et al. (2025). Kramnik mice are well-characterized inbred strains that have been extensively used in pharmacology and lesion-compartment drug-exposure studies of complex inflammatory pathology (Driver et al., 2012). Kramnik mice were used in this study because they are an established animal model in our research group that is highly susceptible to tuberculosis, making them well-suited for ongoing tuberculosis research and for evaluating CBD as an adjunct treatment. Additionally, these mice have been used to successfully administer CBD via a buccal route and to quantify CBD and its major hydroxylated/carboxylated metabolites across different biological matrices using LC-MS/MS, demonstrating the feasibility of CBD metabolism and disposition profiling in this model (Omotayo et al., 2025). Detailed animal husbandry practices have been described in Omotayo et al. (2025). Briefly, 5 mice were randomly selected and administered 10 mg/kg of CBD suspended in sunflower oil intrabuccally, and 5 mice received only the vehicle (10 µL of sunflower oil) intrabuccally (controls). Immediately before compound administration, mice were anesthetized using a VetFlo Anesthesia System (2% isoflurane in 100% O_2_ at a flow rate of 1.5 L/min for 1 min). Isoflurane does not undergo hepatic metabolism; therefore, it is not expected to interfere with the study interventions (Navarro et al., 2021). Anesthetisation ensured stealth of study animals during the administration process, thus ensuring effective dosing. Both the CBD-treated and vehicle control groups received identical daily isoflurane administration; hence, the only variable in this study was CBD. Treatment was administered daily for 2 weeks, and 6 h post dosing on the last day, all mice were humanely sacrificed (mice were exsanguinated by transection of the carotid arteries and jugular veins for blood sampling) to collect tissue samples (brain, liver, and kidney). These tissue samples were rinsed in ice-cold phosphate-buffered saline to remove excess blood, snap-frozen using liquid nitrogen, and stored at −80 °C until use.

### 2.2 Metabolite extraction

A small amount of each tissue was pooled to create a quality control (QC) sample for each tissue type. For sample preparation, 1 g of pre-frozen tissue was weighed into 5 mL microcentrifuge tubes. Metabolites were extracted using a 6:2:2 (v/v) ratio of methanol:water:chloroform mixture, following a slight modification of the Bligh-Dyer extraction procedure (Gullberg et al., 2004). Briefly, each tissue sample was first extracted with 600 µL of methanol and 200 µL of water. Tissue samples were homogenized with 3 mm and 5 mm stainless-steel beads in a vibration mill for 2 min at 30 Hz (MM 400, Retsch). Thereafter, 200 µL of ice-cold chloroform was added, and the steel beads were removed. The samples were vortexed vigorously for 1 min and then placed on ice for 10 min. Thereafter, samples were centrifuged for 10 min at 20,000 × *g* (4 °C). The supernatant (60 µL) was transferred into clean 2 mL screw-top glass vials (Agilent) and carefully dried under a slow, steady stream of nitrogen gas (to avoid oxidation and physical loss of the metabolites) until the solvents were removed.

### 2.3 NMR analysis

NMR analysis followed a miniaturized procedure described by Mason et al. (2018). Briefly, dried extracts were reconstituted with 60 µL of filtered, sterile water and vortexed for 2 min. The mixture was transferred to 2 mL Eppendorf tubes and centrifuged at 12,000 x *g* for 5 min at ambient temperature. The supernatant (54 µL) was mixed with 6 µL of NMR buffer (pH 7.4; internal standard–trimethylsilylpropionic-d4 acid in deuterium oxide) and loaded into a 2 mm glass NMR tube using the eVol NMR digital syringe with a 100 μL syringe and 180 mm-long bevel-tipped needle. Samples were run on a Bruker Avance III HD 500 MHz NMR spectrometer (for all NMR technical settings, see Mason et al., 2018). Samples were analyzed in random order, with a QC sample analyzed at set intervals. Three batches were run for the three tissue types. Variable-sized binning was performed on all discernible peaks in the ^1^H-NMR spectra, and the spectra were normalized to TSP to account for analytical variation.

### 2.4 Statistical analysis

Statistical analysis was performed using MetaboAnalyst (V6.0) (www.metaboanalyst.ca/). Data were normalized by log-transformation and Pareto scaling to generate parametric data, reduce heteroscedasticity, and approximate a normal distribution, while mitigating the influence of high-variance metabolites and preserving relevant biological data for multivariate modeling. Unsupervised principal component analysis (PCA) score plots were generated to assess QCs and the distribution of the experimental data. Supervised partial least squares-discriminant analysis (PLS-DA), including cross-validation and a permutation p-value, was performed to identify variables that may drive differentiation. Fold change, t-test, and effect size were calculated for the non-parametric data for both the control group and tissue samples.

## 3 Results

### 3.1 Quality assurance

High analytical reproducibility and low instrumental drift across each analytical run were evidenced by tightly clustered QC samples in the PCA plots (Supplementary Figure S1 – see Supplementary Material). Clear dispersion among experimental samples indicates genuine biological variation. The PCA models’ performance met recognized validation standards for validity and robustness, as indicated by PERMANOVA p-values <0.05 across 999 permutations, demonstrating strong statistical significance. In summary, analytical variation was negligible, allowing us to focus on biological variation in the CBD-treated and control groups.

**FIGURE 1:**
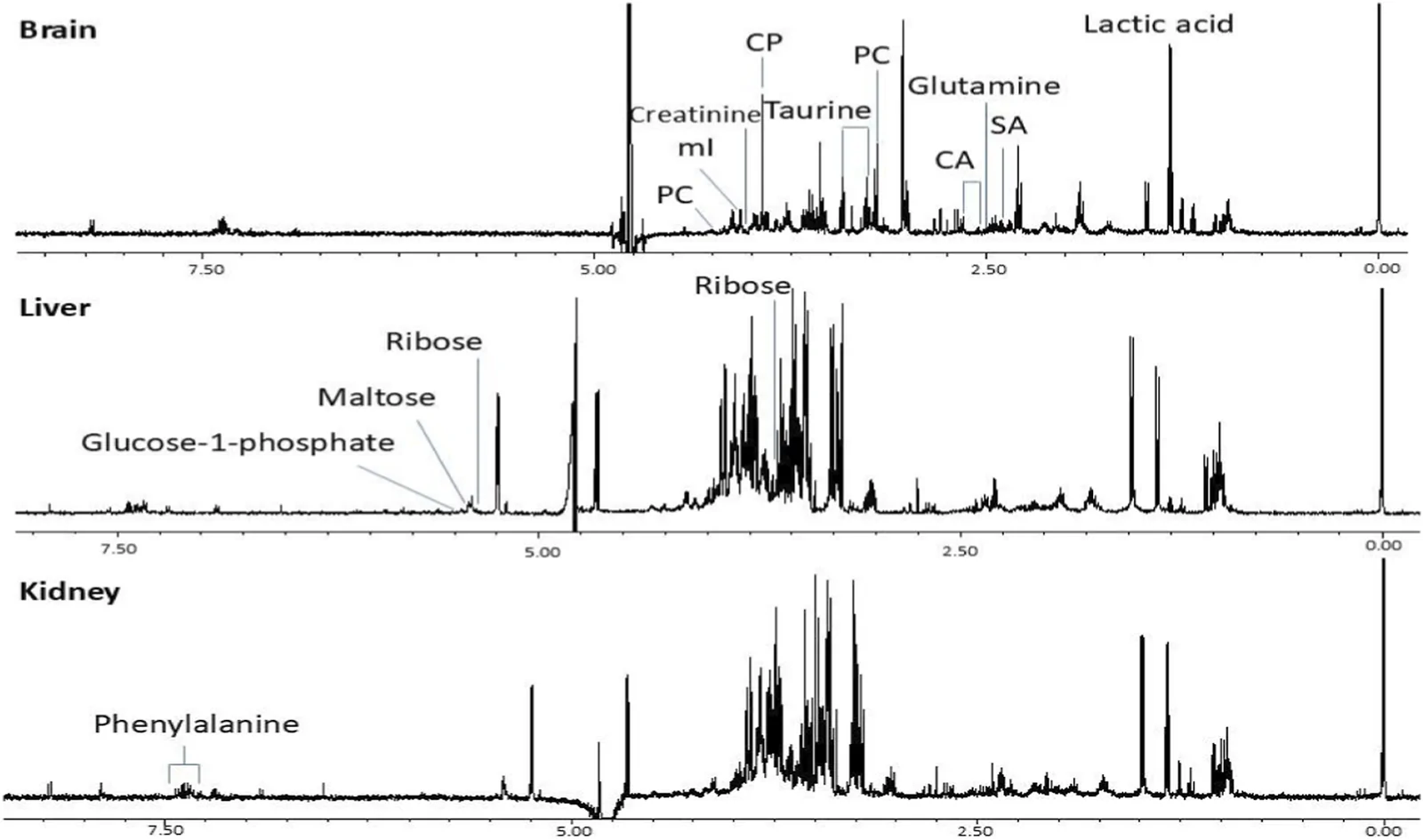
Representative ^1^H-NMR spectra of CBD-treated brain **(A)**, liver **(B)**, and kidney **(C)** tissue QC samples. Significant metabolites are labeled. SA = succinic acid, CA = citric acid, PC = phosphocholine, CP = creatine phosphate, mI = myo-Inositol.

Figure 1 shows representative ^1^H-NMR QC samples for each tissue type: brain (Figure 1A), liver (Figure 1B), and kidneys (Figure 1C). Qualitative evaluation of the ^1^H-NMR spectra reveals numerous discernible peaks with good resolution (<1 Hz peak width). Thus, high-quality ^1^H-NMR spectra were obtained in this study. Notably, the liver (Figure 1B) had the most peaks, as expected given its role as the metabolic hub.

### 3.2 Multivariate statistics results

Unsupervised PCA was conducted to assess and elucidate variable characteristics by comparing variance across the CBD-treated and control groups in liver, kidney, and brain tissue samples. Supervised PLS-DA was performed to maximize covariation and enhance group separation.

**FIGURE 2:**
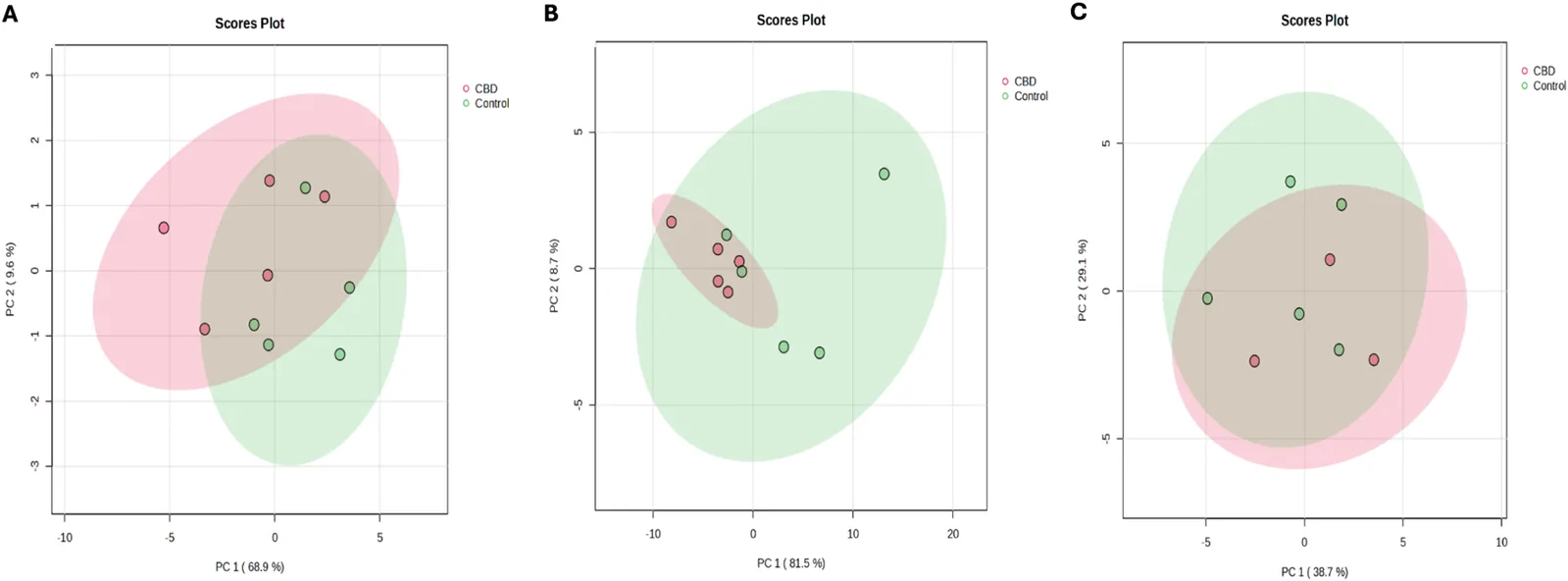
PCA scores plots of CBD-treated (red) and control (green) groups for **(A)** brain, **(B)** liver, and **(C)** kidney tissue samples.

The correlation between tissue samples treated with CBD and the control group was evident in the PCA plots, with PERMANOVA p-values below 0.05 across 999 permutations (Figures 2A–C) and no outliers. In Figure 2A, PC1 accounted for 68.9% of the total variance, and PC2 for 8.6%, indicating clear group separation with a cumulative variance of 77.5%. Control samples were dispersed toward the positive PC1 axis, whereas CBD-treated samples tended to cluster along the negative PC1 axis. This directional divergence suggests significant CBD-induced metabolic changes in brain tissue, despite the slight overlap between confidence ellipses. The greatest group discrimination was observed in liver samples (Figure 2B). The cumulative variance was 90.2%, with PC1 accounting for 81.5% and PC2 for 8.7%. Samples treated with CBD grouped tightly, indicating a steady metabolic response to therapy. Control samples, however, showed a wider dispersion. In kidney samples (Figure 2C), the variance was more evenly distributed between PC1 (38.7%) and PC2 (29.1%), with a total explained variance of 67.8%. Although there was little distinction between the CBD-treated and control groups, the confidence ellipses overlapped more in the kidney than in the brain (Figure 2A).

The PLS-DA model for each tissue type was evaluated using cross-validation metrics (R^2^, Q^2^, and accuracy) and a permutation test. Across all three PLS-DA models, Q^2^ values were below 0.5, indicating low robustness. Moreover, the permutation p-values for each PLS-DA model were greater than 0.05, indicating overfitting, likely due to the small sample size. Hence, the supervised PLS-DA model was not suitable for this data.

### 3.3 Univariate statistical significance

Statistically significant metabolite bins were identified using a volcano plot with log2 fold change >1.5 and p-value <0.1, as well as a medium effect size (g-value >0.6). Based on these univariate cutoffs, the brain had 12 significant ^1^H-NMR bins, followed by 7 from the liver and 1 from the kidney samples (see Supplementary Figures S2-S4 for box plots). Annotation of these significant spectral bins was performed using pure-compound 1D ^1^H-NMR spectral library databases (Table 1); metabolite annotations were confirmed as level 1 using 2D JRES and COSY NMR, and these metabolites were quantified in micrograms per milligram of tissue. Significantly altered metabolites identified in the brain were composed mostly of amino acids and organic acids, while altered sugars were found in the liver samples, and only phenylalanine was found to be altered in the kidney samples. Notably, two significant bins in the brain (3.102 ppm and 5.106 ppm) and one in the liver (7.103 ppm) did not match any spectral library and could not be annotated (unknowns).

**TABLE 1:** Statistically significant bins/metabolites.

Bin (ppm)	Metabolite	Tissue	CBD-treated (mean ± SD)	Control (mean ± SD)	T-test (p-value)	ES (g-value)
2.390	Succinic acid	Brain	10.74 ± 1.75	12.16 ± 2.09	0.279	**0.737**
2.519	Glutamine	Brain	19.12 ± 2.24	27.03 ± 6.64	**0.054**	**1.598**
2.558	Citric acid	Brain	106.46 ± 15.17	119.9 ± 10.91	0.150	**1.017**
3.102	? (unknown)	Brain	​	​	​	​
3.219	Phosphocholine	Brain	24.95 ± 5.41	31.38 ± 2.95	**0.057**	**1.476**
3.254	Taurine	Brain	122.56 ± 16.5	146.96 ± 18.13	**0.057**	**1.408**
3.944	Creatine phosphate	Brain	21.37 ± 3.58	26.92 ± 2.09	**0.022**	**1.897**
4.054	Creatinine	Brain	4.53 ± 1.46	6.75 ± 1.09	**0.028**	**1.727**
4.061	myo-inositol	Brain	171.07 ± 25.17	212.42 ± 18.02	**0.020**	**1.889**
4.099	Lactic acid	Brain	138.01 ± 37.88	164.94 ± 19.9	0.208	**0.890**
4.173	Phosphocholine	Brain	24.95 ± 5.41	31.38 ± 2.95	**0.057**	**1.476**
4.258	Threonine	Brain	33.74 ± 7.17	46.69 ± 7.95	**0.027**	**1.710**
3.600	Ribose	Liver	281.47 ± 60.11	105.86 ± 32.77	**0.001**	**3.627**
5.106	? (unknown)	Liver	​	​	​	​
5.373	Ribose	Liver	281.47 ± 60.11	105.86 ± 32.77	**0.001**	**3.627**
5.416	Maltose	Liver	308.35 ± 61.02	101.06 ± 54.19	**<0.001**	**3.592**
5.460	Glucose-1-phosphate	Liver	175.59 ± 38.68	77.32 ± 38.43	**0.004**	**2.549**
7.103	? (unknown)	Liver	​	​	​	​
8.459	Formic acid	Liver	8.69 ± 4.69	2.66 ± 0.95	**0.044**	**1.779**
7.381	Phenylalanine	Kidney	66.57 ± 2.85	78.69 ± 12.47	**0.096**	**1.175**

p-value <0.1 or g-value >0.6; shown in bold from brain, liver, and kidney tissue samples. Concentrations are given as µg metabolite per mg tissue. SD, standard deviation. ES, effect size.

## 4 Discussion

This study used an untargeted ^1^H-NMR metabolomics approach to investigate tissue-specific (brain, liver, and kidneys) metabolic effects of a 2-week daily CBD treatment in a healthy mouse model. Administration of CBD to these healthy mice showed harmless metabolic stimulation that improves healthy signaling, encouraging a good immune response and energy supply to healthy cells in the tissues. The brain was the most affected organ, with 10 significant metabolites, followed by the liver with 4 and the kidneys with 1. We provide biological context for these results.

### 4.1 Brain

The brain is a high-energy organ in which mitochondria play a vital role in energy supply. The Krebs cycle is compartmentalized in the mitochondrial matrix, and mitochondrial dysfunction can lead to the accumulation of Krebs cycle intermediates–such as succinic acid and citric acid. In an infected brain, activated microglial cells shift from oxidative phosphorylation to an aerobic glycolytic state, thereby inhibiting isocitrate dehydrogenase and transferring citric acid from the mitochondria to the cytosol, thereby influencing the production of fatty acids and inflammatory mediators (nitric oxide and prostaglandin E2). This process regulates and activates the host immune response against infection. Therefore, the decreased presence of citric acid in the brains of CBD-treated mice suggests lower microglial activity and ROS production (Abdel-Salam et al., 2014; Williams and O’Neill, 2018). A study by Pozdnyakov et al. (2021) assessed the effects of succinic acid derivatives on rats with cerebral ischemia and found a significant increase in glycolytic capacity, normalizing anaerobic metabolism (Pozdnyakov et al., 2021). According to Zhang et al. (2024), brain-gut health studies in mice showed that succinic acid induces inflammatory responses in both the intestinal and brain immune systems, underscoring its signaling significance. Another study by Huang et al. (2023) found that cerebral ischemia or reperfusion injury in rats led to elevated plasma and brain tissue succinic acid levels, thereby inhibiting neural stem cells and exacerbating injury. Hence, succinic acid can be considered a signaling molecule that plays an important central role in the host’s inflammatory response. The slight decrease in succinic acid and citric acid in the brains of CBD-treated mice in this study aligns with the anti-inflammatory effects of CBD, suggesting lower ROS levels in these mice.

Glutamine was also slightly decreased in the brains of healthy mice treated with CBD. Glutamine is a vital metabolite for fueling immune cells and is the most supplemented nutrient in the human body to prevent organ damage during critical illnesses, such as sepsis and meningitis (Newsholme et al., 1985; Ardawi, 1988). Glutamine has antioxidant effects, serving as a precursor for glutathione synthesis, the major cellular antioxidant that scavenges free radical species. Glutamine, an immunonutrient, has been shown to provide neuroprotection by inhibiting microglia-mediated neuroinflammatory responses following traumatic brain injury in rats (Huang et al., 2018). Glutamine is also involved in the glutamate-glutamine cycle, in which neurons take up glutamine and convert it to glutamic acid, which is used for glutamatergic synaptic responses. Glutamic acid is an excitotoxic compound. Tissue samples from humans with drug-resistant epilepsy were treated with CBD at various concentrations, which showed a reduction in the release of glutamate at lower concentrations. This was also observed in hippocampal brain tissue from rats with spontaneous recurrent seizures (Martinez-Aguirre et al., 2023). Therefore, in this study, reduced glutamine availability in the brain suggests less glutamic acid release by neurons. Based on this, we suggest that CBD has a neuroprotective effect.

We observed decreased phosphocholine in the brain tissue of CBD-treated mice. The Kennedy pathway is the pathway in which choline undergoes phosphorylation, forming the by-product phosphocholine, which is further converted into the abundant lipid phosphatidylcholine, which is essential for brain integrity and immunity (Kennedy and Weiss, 1956). The brain is a lipid-rich organ, and phosphocholine is an indicator of healthy, sustained membrane synthesis (Thompson et al., 1999; Taghibiglou and Khalaj, 2017). A healthy brain cell membrane was found to play a vital role in neurotherapy and drug delivery in an *in vivo* study using nanoparticle therapy in Sprague-Dawley rats (Han et al., 2021). A study by Bernhard et al. (2022) reported the essential role of phosphocholine in the development of both human and rodent infants, confirming its role in maintaining cell membrane integrity. Hence, the lower levels of phosphocholine in the brain after CBD treatment in this study are in line with the CBD mechanism of action, which modulates phospholipid metabolism and influences cell membrane health and immune response.

Taurine is an amino acid that has been shown to regulate neuroprotection, antioxidant defense, neurotransmission inhibition, and osmoregulation (Schaffer et al., 2010; Jakaria et al., 2019; Rafiee et al., 2022). Our study found slightly lower taurine levels in the brains of healthy mice treated with CBD. CBD has been shown to exhibit neuroprotective synergy with brain metabolites (Kimura et al., 2019; Peres et al., 2018). Hence, we postulate that the brain needs less taurine when another neuroprotective agent (CBD) is available. Threonine was also slightly lower in the brains of CBD-treated mice. Threonine is a precursor to glycine, an inhibitory neurotransmitter and a co-agonist of the N-methyl-d-aspartate (NMDA) receptor in the brain. Threonine is also an essential amino acid that is both glucogenic and ketogenic. Hence, the observed lower threonine levels suggest reduced signaling and potentially lower brain activity, possibly due to a more metabolically balanced state induced by CBD. Myo-inositol is a cyclic sugar alcohol that is abundant in the brain and regulates the formation of neurotransmitters and phosphatidylinositol signaling systems. Lower myo-inositol levels support the notion that signaling in the brain is reduced by CBD treatment.

Creatinine is a by-product of the non-enzymatic breakdown of creatine and creatine phosphate in animals and serves as a biomarker of kidney and muscle health, as well as degenerative disease (Smith-Palmer, 2002; Mobisson et al., 2023). Creatine phosphate is a high-energy reserve metabolite that provides rapid energy to cells in the muscle and brain, which require a swift energy supply (Peres et al., 2018; Hong and Huang, 2025). According to Valvassori et al. (2013), acute and chronic intracerebral injection of CBD increased creatine kinase activity in brain tissue. Animal model studies have shown that CBD positively influences energy metabolism, which is closely linked to creatine phosphate. However, we found no studies examining the effects of creatinine and creatine metabolite characteristics in brain tissue samples from rodents or humans. The lower levels of lactic acid in the brains of the CBD-treated mice also support the hypothesis of lower neuroenergetics.

### 4.2 Liver and kidneys

The CBD-treated mice in this study exhibited increased levels of carbohydrates, specifically ribose, maltose, and glucose-1-phosphate. Ribose is a simple monosaccharide, and in animals, its presence and metabolic flux in the liver indicate that CBD modulates energy production, antioxidant activity, and cell viability. A study by Sun et al. (2017) found that CBD stimulates the pentose phosphate pathway (PPP) by increasing glucose-6-phosphate dehydrogenase (G6PD) activity, thereby providing antioxidant protection against oxygen-glucose deprivation. A study by Erukainure et al. (2022) supports the idea that ribose presence regulates the PPP, thereby generating antioxidant activity in the liver. A study by Carter (2022) demonstrated the therapeutic effects of CBD without harming the host and showed that higher doses of CBD induced cleavage of poly(ADP-ribose) polymerase (PARP-1), which regulates DNA repair and replication. Therefore, we suggest that CBD increases energy supply and improves liver cell health. Maltose is a disaccharide consisting of two glucose molecules and is catabolized by alpha-glucosidase. The accumulation of maltose suggests that CBD may inhibit alpha-glucosidase. There was also an observed shift in glycolytic flux, with increased glucose-1-phosphate levels in the liver of healthy CBD-treated mice. The reduced breakdown of sugars and accumulation of the glycolytic intermediate glucose-1-phosphate indicate reduced carbohydrate utilization for energy production.

The kidney tissue samples showed minimal differences between the two experimental groups, with only a slight decrease in phenylalanine concentration in the CBD-treated healthy mice. Phenylalanine is an amino acid involved in protein synthesis and a precursor to tyrosine, which is used in the synthesis of some neurotransmitters (e.g., dopamine and serotonin). Phenylalanine is also a marker positively correlated with stress (Hüfner et al., 2020). Hence, reduced levels of phenylalanine in the kidneys of the CBD-treated mice suggest less renal stress. A study by Baban et al. (2018) concluded that CBD has renoprotective properties. Therefore, CBD has a positive effect on kidney health.

## 5 Conclusion

**FIGURE 3:**
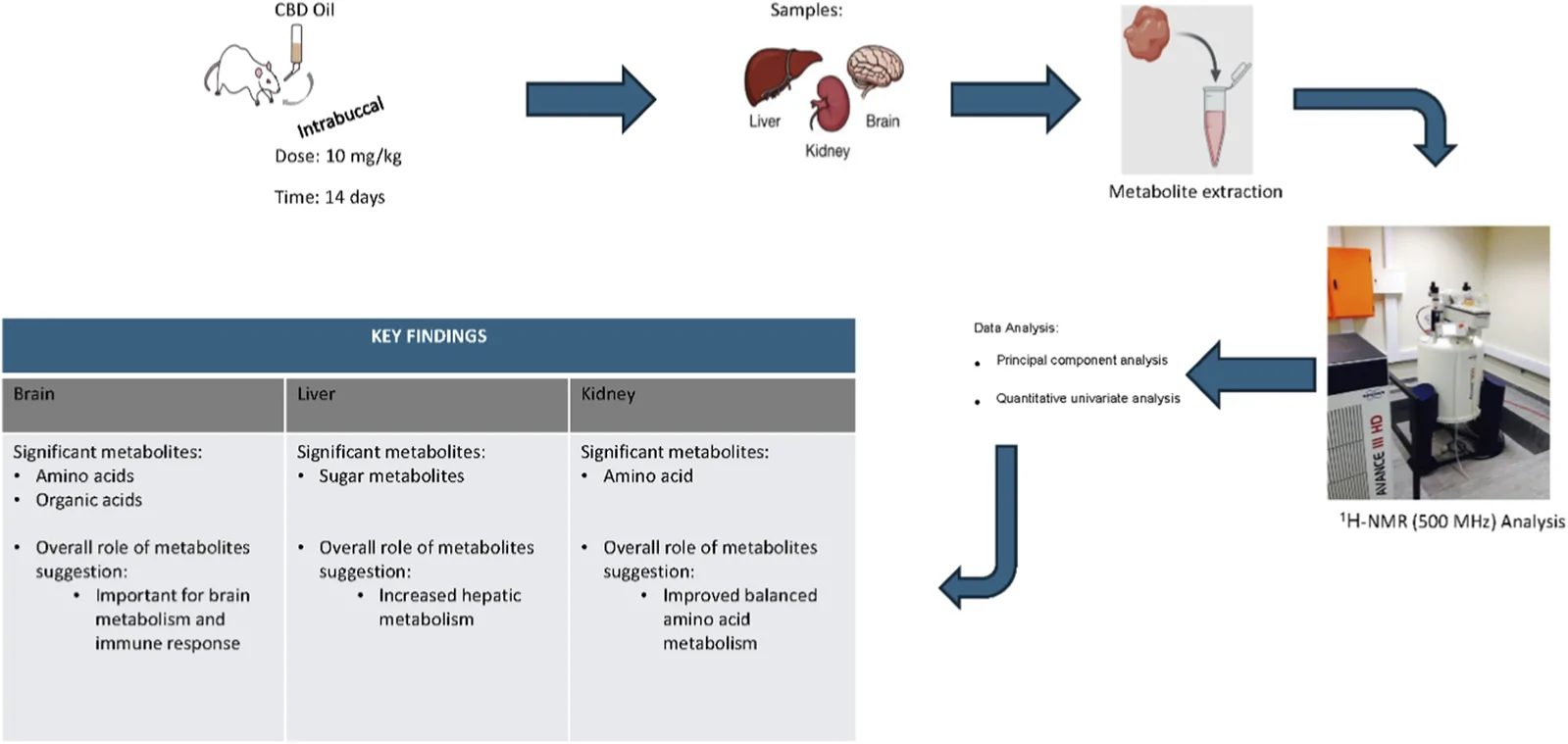
Graphical summary of the experimental design and results of this study.

Based on these tissue-specific untargeted ^1^H-NMR results, CBD does not appear to have any discernible adverse effects on the metabolism of these mice, despite their smaller size and higher basal metabolic rate, which make them more susceptible to metabolic suppression than humans. This untargeted ^1^H-NMR metabolomics study allowed us to generate a hypothesis. More sensitive metabolomics methods (e.g., GC-MS or LC-MS) that focus on the metabolite classes described in this study are recommended. The small sample size of this study is another limitation, given the natural heterogeneity of mouse samples, which can lead to false discoveries and model overfitting. However, this study aligns with numerous other studies showing that CBD is therapeutic and has negligible harmful side effects. Indeed, a larger cohort is needed to confirm these results. From a metabolic perspective, we speculate that CBD could be used safely as an adjunctive treatment for systemic inflammation and oxidative stress in inflammatory disease states. This conclusion is based on the absence of harmful biological triggering metabolites detected in this study. It should also be noted that this study focused on the metabolic influence of CBD in these tissues in the absence of any pre-existing pathology, to ascertain that the observed effects indeed result from administration of CBD, not a disease. A graphical summary of the experimental design and results of this study is given in Figure 3.

## Data Availability

The raw data supporting the conclusions of this article will be made available by the authors, without undue reservation.
