# Supplementary material for: Tissue-specific untargeted 1H-NMR metabolomics reveals no adverse metabolic effects of daily intrabuccal cannabidiol (CBD) in healthy mice
Source: Front Pharmacol. 2026 Aug 20;17:1896842. doi: 10.3389/fphar.2026.1896842 (PMC13537750; doi:10.3389/fphar.2026.1896842)
Supplement: Supplementary file 1 [file Supplementaryfile1.docx]

**A**

**B**

**C**

Figure S1: PCA plots of Brain (A), Liver (B), and Kidney (C) tissue samples show that QC samples (blue) cluster together, indicating reliable data.

Figure S2: Brain tissue significant bins box plots.

Figure S3: Liver tissue significant bins box plots

Figure S3: Kidney tissue significant bin box plot.
